# The relationship between obesity, diabetes, hypertension and vitamin D deficiency among Saudi Arabians aged 15 and over: results from the Saudi health interview survey

**DOI:** 10.1186/s12902-020-00562-z

**Published:** 2020-06-05

**Authors:** Ibrahim Al-Sumaih, Brian Johnston, Michael Donnelly, Ciaran O’Neill

**Affiliations:** 1 Centre for Public Health, School of Medicine, Dentistry and Biomedical Sciences, Queen’s University Belfast, Belfast, UK; 2 Belfast Health and Social Care Trust, Belfast, UK; 3 Institute of Clinical Sciences, Block B, Royal Victoria Hospital, Belfast, BT12 6BA UK

**Keywords:** Essential hypertension, Diabetes mellitus type 2, Metabolic syndrome, Obesity, Dietary supplements, Sedentary behaviour

## Abstract

**Background:**

Obesity and diabetes are related conditions, the prevalence of which has increased globally in recent years. These conditions have been linked to hypertension and vitamin D deficiency though the nature of the relationship remains unclear and is likely to vary between identifiable groups and specific contexts. The aim of this paper is to examine the relationships between obesity, type 2 diabetes, hypertension and vitamin D, among Saudis citizens aged 15 and over.

**Methods:**

Self-reported and measured data were taken from the 2013 Saudi Health Interview Survey and analysed using a series of seemingly unrelated bivariate probit regression (SURBVP) analyses. Sensitivity analyses were undertaken in which the selection and specification of covariates and outcomes were varied.

**Results:**

In the main analysis data on 957 women and 1127 men were analysed. Differences were evident between men and women in the prevalence of type 2 diabetes, obesity, central obesity, hypertension and vitamin D deficiency. While men were more likely to experience diabetes and hypertension, women were more likely to experience obesity, central obesity and vitamin D deficiency. In multivariable analyses obesity and age were found to significantly predict hypertension risk in women; central obesity to predict diabetes risk in men and women, as well as hypertension risk in men. Vitamin D was not found to predict diabetes risk nor hypertension risk in either sex. Milk consumption and sun exposure were found to predict vitamin D deficiency in women but not men. While there was evidence of unobserved heterogeneity in models predicting diabetes and hypertension, there was no evidence of unobserved heterogeneity between these and those predicting vitamin D deficiency. Results did not materially change over a range of sensitivity analyses.

**Conclusion:**

While there is strong evidence of distinct patterns in the relationship between diabetes, hypertension and obesity among men and women in Saudi Arabia and in the risk of vitamin D deficiency, we found no evidence of a relationship between vitamin D levels and risk of either diabetes or hypertension.

## Background

The prevalence of the closely related conditions of obesity and diabetes has increased globally in recent years [1]. Together they place a significant burden on healthcare systems. For example, in the USA in 2010, obesity and diabetes consumed an estimated 20% of total healthcare expenditure, while in Saudi Arabia in the same year, diabetes alone consumed 21% of healthcare resources [2]. There is an urgent need to improve our understanding of the complex relationship between obesity, diabetes and related conditions such as cardiovascular disease in order to develop and implement more effective policy responses. The relationships between the conditions and the ensuing burden vary between populations according to their social norms, diets, climates and healthcare systems. This variation points to the importance of taking account of the *context* in which these relationships function to improve our understanding about the potential for policy interventions. Moreover, the existence of heterogeneity at an individual level in relation to observable characteristics such as age and gender and unobservable characteristics such as genetic susceptibility or unobserved behaviours adds a further layer of complexity.

Research suggests the existence of a physiological relationship between obesity, diabetes, levels of vitamin D and hypertension. Low levels of serum vitamin D have been associated with increased risk of type 2 diabetes [3] and with increased risk of hypertension [4]. A negative association between levels of vitamin D and BMI has been found whilst conversely a positive association has been observed between risk of type 2 diabetes and BMI [5–7]. According to existing research, the interpretation of the nature, direction and meaning of these relationships is complex and equivocal. For example, different patterns of the relationship between vitamin D and hypertension have been observed across races and genders [4, 8]; while the results of recent randomised controlled trials suggest that any benefit to blood pressure associated with vitamin D sufficiency may relate to factors other than vitamin D per se [9].

In countries such as those in the Middle East where a literature points to the existence of high levels of vitamin D deficiency particular to specific groups [10] it is possible that the analysis of relationships may afford insights into the role of specific characteristics and behaviours. For example, studies conducted in Arabic countries have reported the existence of differences in vitamin D levels between men and women [11, 12] between women who wear and those who do not wear hijab [13] and among men and women of different ages [12]. Associations have also been noted in this context in respect of use of vitamin D supplements, milk consumption and measures of central obesity [12]. The existence of large numbers of people with low but heterogeneous levels of vitamin D related to observable characteristics may thus provide an opportunity to examine relationships between obesity, diabetes and vitamin D deficiency adjusting for these characteristics and behaviours as well as disease status for comorbid conditions such as hypertension. Whether these associations are direct – as between obesity and diabetes risk – or indirect - as between vitamin D and diabetes or hypertension – they may nevertheless offer potentially useful policy insights.

The aim of this study is to examine the relationships between type 2 diabetes, vitamin D deficiency, hypertension status and different measures of obesity controlling for a range of observable characteristics thought to affect these relationships using data from the Saudi Health Interview Survey (SHIS) [14]. In addition, our analytic approach seeks to leverage data related to the residuals in our fitted models in order to incorporate unobserved heterogeneity into our analysis. This is the first study to the best of our knowledge to examine these relationships collectively and to adopt an analytic approach that involves leveraging potentially useful data related to unobserved heterogeneity within this particular geographic context.

## Methods

Data were extracted from the Saudi Health Interview Survey (SHIS) for 2013 on 957 females and 1127 males. The SHIS is a multistage survey of individuals aged 15 years and older [15–17]. As the data is anonymized, and it is open access, with general consent taken during the survey, no ethical approval was required for use of the data.

Using a nationally representative randomly drawn sample, the survey collects data on a range of socio-demographic characteristics such as age, gender, educational attainment, marital status and income along with physical data on blood pressure, height, weight and blood samples, self-reported diet and physical activity. In this study, we extracted from SHIS on measured height and weight, serum vitamin D levels (concentration of serum 25(OH)D), self-reported hypertension and type 2 diabetes status as well as a range of characteristics that included age, income, time spent in sedentary activities (hours spent sitting per day), consumption of diary products, smoking status and exposure to sunlight. Measured HbA1c and blood pressure were also extracted for use in sensitivity analyses. Outcomes related to vitamin D deficiency status (a dichotomous variable equal to 1 if serum vitamin D levels were below 20 ng/mL and zero otherwise), type 2 diabetes status (a dichotomous variable equal to 1 if the individual reported having type 2 diabetes and zero otherwise) and hypertension status (a dichotomous variable equal to 1 if the individual reported having hypertension, and zero otherwise.) The latter reflected the inclusion of hypertension as an indicator of cardiovascular disease in the analysis.

Covariates used to explain the risk of these outcomes were obesity status (a dichotomous variable equal to 1 if the individual’s measured BMI was greater than or equal to 30 and zero otherwise); central obesity (a dichotomous variable equal to 1 if the individual’s measured waist circumference was equal to or greater than 88 cm for female or 102 for male, zero otherwise); age (in years), milk consumption (a dichotomous variable equal to 1 if the individual consumed milk once or more per week, zero otherwise); the number of hours spent sitting reported by the individual per day), exposure to sunlight (a dummy variable equal to one if the individual spent at least 25 min per day in direct sunlight and zero otherwise), whether the individual has ever smoked (a dummy variable equal to 1 if the individual had ever smoked and zero otherwise) and; the individual’s income (a dichotomous variable equal to 1 if the individual’s monthly household income was reported to be 15,000 Riyals, or above, equivalent to $4000 US dollars,). SHIS participants diagnosed to have type 1 diabetes and those who consumed a vitamin D supplement as well as those for whom data was absent for the included variables were omitted from analyses. Type 1 diabetics were excluded as this was considered to be a distinct disease to type 2 diabetes with distinct relationships with obesity and other covariates in the analysis. Those who consumed vitamin D supplements were also excluded from the main analyses. Unlike hypertension and diabetes where medicines may be prescribed to those with the condition in a controlled manner, vitamin D supplements are freely available over the counter. Not only may their use be more ubiquitous therefore but the dose consumed is also more likely to be uniformed by medical advice and to exhibit significant variation unrelated to health. Rather than introduce this additional heterogeneity into the analysis this group were excluded to be examined in further research.

A descriptive analysis of the data was first undertaken in which continuous variables were described using mean and standard deviations and dichotomous variables as percentages. Differences in means between groups related to age and vitamin D levels were estimated and examined for significance. Three multivariable probit models were estimated one for each outcome as a function of specific covariates using probabilistic multivariable regression analyses. Hypertension status was specified as a function of age, obesity, central obesity, vitamin D level, income and whether the individual had ever smoked. Diabetes status was specified as a function of age, obesity, central obesity and income. The choice of covariates were informed by relationships reported in the literature in respect of age and obesity/distribution of body fat in the case of diabetes and hypertension [18, 19]. Both income and smoking status were taken as indicators of health-related behaviours – those who had ever smoked being assumed to being more likely to adopt behaviours consistent with attachment of a lower value to health in general – and those with higher income being more likely to adopt behaviours consistent with a higher value to health in general [20, 21]. Vitamin D status was specified as a function of age, obesity, central obesity, milk consumption, exposure to sunlight and income. Milk consumption and sunlight were included given their physiological potential to affect observed levels of vitamin D. Income was included given its potential association with other health behaviours. Separate models were estimated for males and females to account for the existence of potentially distinct relationships between outcomes and covariates based on gender.

To allow for the possibility of unobserved heterogeneity, related for example to how long a condition had been experienced, family history or unobserved behaviours, the models were estimated using a series of seemingly unrelated bivariate probit models (SURBVP) [22]. In the SURBVP model related outcomes are specified as functions of covariates that may or may not be identical. The functions are estimated simultaneously and any correlation in errors used to test for and take account of possible unobserved heterogeneity. Thus, in the presence of unobserved heterogeneity a distinct pattern in residuals across functions would be observed that is informative. For example, if vitamin D does improve the body’s sensitivity to insulin and thus reduce the risk of type 2 diabetes, were we to under-predict the likelihood of vitamin D deficiency due the exclusion of some unobserved attribute or behaviour (for example those who adopt preventive measures to protect health such as the use of sun screen) we might also over-predict the likelihood of type 2 diabetes due to exclusion of the same factor (use of preventive measures, related for example to diet). Thus, a negative correlation in errors would be observed. Uncorrected this would result effectively in omitted variable bias in the production of estimated relationships. The SURBVP adjusts for this using the variance covariance matrix from the seemingly unrelated regressions. Using the *mvp* command in STATA 15, the model allows for estimation across three or more functions simultaneously [22]. The inclusion of vitamin D levels as a covariate and vitamin D deficiency as an outcome reflects the potentially endogenous nature of vitamin D in the model, that is, as something that is both determined by behaviours like milk consumption, as well as potentially determining outcomes like diabetes risk. Deficiency status as opposed to levels was used as an outcome to permit use of the bivariate approach which requires that the outcomes compared are dichotomous.

A series of sensitivity analyses were undertaken as part of the analysis. First, we varied covariates included in the models, including the number of hours spent sitting and varying the levels of sunlight used to define adequate exposure to sunlight. The number of hours spent sitting was included in the models for diabetes and hypertension risk based on reported relationships between sedentary behaviours and risk of these conditions [23].

Second, the analyses were re-estimated in which individuals with a measured HbA1c above 6.5% and a measured blood pressure in excess of 140/90 mmHg were included in addition to those with self-reported diabetes status and hypertension status respectively to take account of potential undiagnosed disease. With respect to hypertension an average blood pressure in excess of 140/90 mmHg taken over three measures was used to define measured hypertension. Individuals in this category who had not self-reported hypertension were added were added to the self-reporting group.

Third, as use of prescribed medication may affect observed relationships, the model with undiagnosed participants added to the self-reporting group was extended to include use of medication as covariates. SHIS offer limited information on use of prescribed medicines. However, it was possible to include information on whether a person reported use of medicines for diabetes and cholesterol reduction. No information on use of medicines for hypertension were available in the data. This analysis was only possible for a sub-set of the data on whom medications were reported.

Fourth, we used different cut-off points to define vitamin D deficiency to reflect uncertainty around how deficiency is defined. This involved re-estimating the base model but re-specifying the thresholds used to define vitamin D deficiency. Models were estimated where a threshold of 12 ng/mL was used [24] and of 10 ng/mL [25] were used.

Fifth, we varied the definition of sunlight exposure from 25 down to 10 and up to 30 min per day. In each instance we examined whether our results remained robust to variations in the approach used.

In addition to differences across groups in means/proportions, the sign and significance of covariates in regression analyses, attention was given to the sign and significance of Rho – the correlation in errors across regression models. Where a significant Rho was recorded the importance of incorporating unobserved heterogeneity into the models was supported; where this was insignificant the use of unrelated models was supported.

Models were partitioned based on gender to allow for the possibility of distinct relationships in men and women between outcomes and covariates.

## Results

In Table 1, descriptive statistics for the sample with respect to each outcome and covariate are presented. As can be seen, differences are evident between the genders. Among women, 39.5% of the female sample were obese, 9.5% were hypertensive, 7.6% were type 2 diabetic and 31.5% were deficient in vitamin D. This compares with 31.6% of men being obese, 12.4% hypertensive, approximately 12.5% diabetic and 13.7% being deficient in vitamin D at the 20 ng/mL concentration of serum 25(OH) D threshold. The differences in vitamin D levels are significant and differences in disease levels suggestive of distinct risk profiles indicating distinct risk profiles across genders. In Table 2 the results of the base case mvp probit analysis are presented. As can be seen, waist circumference is predictive of type 2 diabetes in females, while BMI-based obesity is predictive of hypertension. Among men, waist circumference is predictive of type 2 diabetes risk while waist circumference and BMI-based obesity are predictive of hypertension and vitamin D deficiency. Interestingly, milk consumption and sunlight exposure are not predictive of vitamin D deficiency in men but are strongly predictive of vitamin D deficiency in women. (While significant differences existed between men and women in respect of exposure to sunlight as reported in Table 1, there were no such differences in respect of milk consumption.) In both genders age is predictive of diabetes and hypertension status.

**Table 1:** Descriptive statistics of the sample

	Female (*n* = 957)		Male (*n* = 1127)			Total (*n* = 2084)		
Variable	Mean	SD	Mean	SD	MD	Mean	SD	*P*-value
Age (years)	37.76	15.13	40.45	17.37	**−2.68**	**39.22**	**16.43**	**0.000**
Vitamin D level (ng/dL)	30.85	19.17	37.20	19.01	**−6.35**	**34.28**	**19.34**	**0.000**
	Frequency	Percent	Frequency	Percent		Frequency	Percent	
Diabetes type 2	73	**7.63**	141	**12.51**		214	10.27	**0.000**
Hypertension	91	**9.51**	140	**12.42**		231	11.08	**0.035**
Vitamin D deficiency	301	**31.45**	154	**13.66**		455	21.83	**0.000**
BMI-based obesity	378	**39.5**	356	**31.59**		734	35.22	**0.000**
Central obesity	495	**51.72**	361	**32.03**		856	41.07	**0.000**
High income	94	**9.82**	164	**14.55**		258	12.38	**0.001**
Ever smoker	10	**1.04**	343	**30.43**		353	16.94	**0.000**
Milk consumption	790	82.55	912	80.92		1702	81.67	0.339
Sun exposure for 25 mins	90	**9.4**	331	**29.37**		421	20.20	**0.000**

figures in bold are significant at *p* < 0.05

**Table 2:** Result of multivariable probit model analysis. (*n* = 957 female and 1127 male)

	Diabetes		Hypertension		Vitamin D deficiency	
	Female	Male	Female	Male	Female	Male
Age	**0.098 (0.009)**	**0.147 (0.000)**	**0.188 (0.000)**	**0.090 (0.001)**	−0.024 (0.060)	− 0.015 (0.260)
Age^2	−0.001 (0.080)	**− 0.001 (0.000)**	**− 0.001 (0.000)**	− 0.000 (0.094)	0.000 (0.244)	0.000 (0.850)
Obesity	0.085 (0.564)	0.217 (0.095)	**0.298 (0.035)**	**0.587 (0.000)**	0.074 (0.489)	**0.247 (0.026)**
Central obesity	**0.602 (0.001)**	**0.333 (0.008)**	0.127 (0.441)	**0.287 (0.036)**	0.138 (0.208)	**−0.234 (0.048)**
High income	**0.436 (0.035)**	0.164 (0.257)	−0.349 (0.158)	0.069 (0.668)	−0.099 (0.518)	0.046 (0.735)
Vitamin D level	0.005 (0.127)	0.004 (0.220)	−0.001 (0.725)	0.003 (0.314)	–	–
Ever smoker	–	–	0.546 (0.283)	−0.095 (0.448)	–	–
Milk consumption	–	–	–	–	**−0.353 (0.001)**	−0.021 (0.858)
Sun exposure	–	–	–	–	**− 0.452 (0.008)**	− 0.006 (0.953)
Constant	**−5.257 (0.000)**	**−6.064 (0.000)**	**−7.144 (0.000)**	**−4.979 (0.000)**	0.392 (0.142)	**−0.573 (0.041)**
Rho Diabetes	1.000	1.000	**0.274 (0.003)**	**0.205 (0.006)**	0.059 (0.431)	−0.016 (0.847)
Rho Hypertension	**0.274 (0.003)**	**0.205 (0.006)**	1.000	1.000	0.057 (0.481)	0.011 (0.877)
Rho Vitamin D deficiency	0.059 (0.431)	−0.016 (0.847)	0.057 (0.481)	0.011 (0.877)	1.000	1.000

Note: *p* value is reported in parenthesis, figures in bold are significant at *p* < 0.05

Rho, which reflects the correlation in errors between models is significant and positive with respect to models estimated for diabetes and hypertension for both males and females. It is significant for neither gender in respect of vitamin D deficiency when examined jointly with either diabetes or hypertension. In Table 3 the results for a vitamin D deficiency model estimated independent of the models for diabetes and hypertension (based on findings with respect to Rho) are reported. As can be seen minimal differences are evident to the results reported in Table 2 with respect to vitamin D deficiency.

**Table 3:** Result of probit model analysis with vitamin D deficiency as an outcome (*n* = 2084)

	Female	Male
Age	− 0.025 (0.056)	− 0.015 (0.277)
Age^2	0.000 (0.231)	0.000 (0.882)
Obesity	0.076 (0.473)	**0.249 (0.024)**
Central obesity	0.133 (0.223)	**−0.235 (0.047)**
High income	−0.099 (0.519)	0.046 (0.734)
Milk consumption	**−0.356 (0.001)**	− 0.021 (0.851)
Sun exposure	**−0.455 (0.008)**	− 0.005 (0.973)
Constant	0.403 (0.131)	**−0.573 (0.036)**

Note: * *p* value is reported in parenthesis, figures in bold are significant at *p* < 0.05

In sensitivity analyses, reported in Table 4, the inclusion of additional covariates related to sedentary behavior (number of hours spent sitting) are shown. As can be seen this did not materially affect the relationships with respect to diabetes, where sedentary behavior was significant among women but not men, though high income was no longer significant in women as a predictor. With respect to hypertension, sedentary behavior was not significant in either males or females, its inclusion reducing the significance of obesity in predicting hypertension status in women but leaving results otherwise largely unaffected. Errors remained positively correlated between the estimated functions for hypertension and type 2 diabetes for both men and women throughout.

**Table 4:** The effect of adding sedentary behaviour to the multivariable probit model. (*n* = 864 female and 1068 male)

	Diabetes		Hypertension		Vitamin D deficiency	
	Female	Male	Female	Male	Female	Male
Age	**0.104 (0.007)**	**0.147 (0.000)**	**0.187 (0.000)**	**0.103 (0.000)**	−0.024 (0.080)	−0.015 (0.254)
Age^2	−0.001 (0.052)	**−0.001 (0.000)**	**− 0.001 (0.000)**	**−0.001 (0.032)**	0.000 (0.263)	0.000 (0.814)
Obesity	0.106 (0.492)	0.197 (0.145)	0.286 (0.060)	**0.526 (0.000)**	0.109 (0.329)	**0.245 (0.031)**
Central obesity	**0.643 (0.001**)	**0.279 (0.033)**	0.102 (0.551)	**0.307 (0.028)**	0.089 (0.435)	**−0.246 (0.042)**
High income	0.398 (0.068)	0.198 (0.181)	−0.462 (0.084)	0.041 (0.805)	−0.061 (0.696)	0.046 (0.740)
Vitamin D level	0.003 (0.340)	0.005 (0.073)	−0.002 (0.563)	0.003 (0.342)	–	–
Ever smoker	–	–	0.783 (0.170)	−0.117 (0.352)	–	–
Milk consumption	–	–	–	–	**−0.368 (0.001)**	−0.024 (0.845)
Sun exposure	–	–	–	–	**−0.416 (0.016)**	0.008 (0.940)
Sitting hours	**0.054 (0.006)**	0.028 (0.082)	0.015 (0.429)	0.028 (0.080)		
Constant	**−5.639 (0.000)**	**−6.218 (0.000)**	**−7.154 (0.000)**	**−5.339 (0.000)**	0.379 (0.176)	−0.554 (0.051)
Rho Diabetes	1.000	1.000	**0.219 (0.017)**	**0.284 (0.000)**	0.026 (0.744)	0.071 (0.354)
Rho Hypertension	**0.219 (0.017)**	**0.284 (0.000)**	1.000	1.000	0.144 (0.089)	−0.028 (0.700)
Rho Vitamin D deficiency	0.026 (0.744)	0.071 (0.354)	0.144 (0.089)	−0.028 (0.700)	1.000	1.000

Note: *p* value is reported in the parenthesis, figures in bold are significant at *p* < 0.05

In further sensitivity analyses reported in Tables 5 and 6 the use of measured in addition to self-reported measures of diabetes and hypertension status and this model extended to include use of prescribed medicines are reported respectively.

**Table 5:** Result of multivariable probit model analysis where diabetes defined as HbA1c > =6.5 and hypertension defined as systolic blood pressure > =140 mmHg or diastolic blood pressure > =90 mmHg or both. (*n* = 1054 female and 1191 male)

	Diabetes		Hypertension		Vitamin D deficiency	
	Female	Male	Female	Male	Female	Male
Age	0.014 (0.311)	**0.028 (0.023)**	**0.059 (0.000)**	**0.037 (0.003)**	−0.016 (0.200)	−0.019 (0.129)
Age^2	0.000 (0.761)	−0.000 (0.589)	−0.000 (0.081)	− 0.000 (0.607)	0.000 (0.700)	0.000 (0.489)
Obesity	0.177 (0.095)	**0.299 (0.004)**	0.183 (0.113)	**0.358 (0.000)**	0.031 (0.763)	**0.293 (0.009)**
Central obesity	0.060 (0.584)	0.022 (0.839)	−0.071 (0.564)	0.120 (0.242)	0.127 (0.228)	**−0.289 (0.015)**
High income	0.123 (0.403)	0.177 (0.124)	−0.214 (0.228)	−0.009 (0.940)	− 0.039 (0.786)	0.042 (0.749)
Vitamin D level	0.001 (0.588)	0.003 (0.245)	0.003 (0.233)	−0.000 (0.984)	–	–
Ever smoker	–	–	0.227 (0.568)	0.086 (0.346)	–	–
Milk consumption	–	–	–	–	**−0.294 (0.004)**	−0.048 (0.677)
Sun exposure	–	–	–	–	**−0.466 (0.004)**	−0.001 (0.994)
Constant	**−1.674 (0.000)**	**−2.098 (0.000)**	**−2.996 (0.000)**	**−2.313 (0.000)**	0.206 (0.422)	−0.506 (0.059)
Rho Diabetes	1.000	1.000	0.034 (0.573)	0.053 (0.305)	−0.069 (0.265)	−0.035 (0.609)
Rho Hypertension	0.034 (0.573)	0.053 (0.305)	1.000	1.000	0.082 (0.204)	0.030 (0.660)
Rho Vitamin D deficiency	−0.069 (0.265)	−0.035 (0.609)	0.082 (0.204)	0.030 (0.660)	1.000	1.000

Note: *p* value is reported in the parenthesis, figures in bold are significant at *p* < 0.05

**Table 6:** Result of multivariable probit analysis controlling for medications (*n* = 2084)

	Diabetes		Hypertension		Vitamin D deficiency	
	Female	Male	Female	Male	Female	Male
Age	**0.104 (0.001)**	**0.169 (0.000)**	**0.186 (0.000)**	**0.088 (0.001)**	−0.025 (0.058)	−0.015 (0.259)
Age^2	**−0.001 (0.017)**	**−0.001 (0.000)**	**− 0.001 (0.000)**	−0.000 (0.096)	0.000 (0.239)	0.000 (0.850)
Obesity	0.138 (0.417)	0.131 (0.367)	**0.310 (0.030)**	**0.557 (0.000)**	0.076 (0.478)	**0.244 (0.027)**
Central obesity	**0.540 (0.008)**	**0.139 (0.023)**	0.081 (0.624)	**0.278 (0.042)**	0.131 (0.236)	**−0.231 (0.049)**
High income	0.301 (0.185)	0.278 (0.075)	−0.390 (0.127)	0.080 (0.619)	−0.091 (0.552)	0.045 (0.740)
Vitamin D level	0.006 (0.104)	0.001 (0.639)	−0.002 (0.670)	0.003 (0.379)	–	–
Ever smoker	–	–	0.562 (0.273)	−0.098 (0.432)	–	–
Milk consumption	–	–	–	–	**−0.350 (0.001)**	−0.025 (0.835)
Sun exposure	–	–	–	–	**−0.455 (0.007)**	−0.007 (0.945)
Hypoglycemic agents	**2.467 (0.000)**	**2.414 (0.000)**	**0.556 (0.037)**	0.335 (0.055)	−0.070 (0.790)	−0.029 (0.894)
Anti-lipidemic medications	−0.164 (0.814)	−0.435 (0.329)	0.229 (0.678)	0.372 (0.186)	0.438 (0.285)	0.005 (0.988)
Constant	**−5.540 (0.000)**	**−6.730 (0.000)**	**−7.066 (0.000)**	**−4.876 (0.000)**	0.393 (0.143)	**−0.569 (0.043)**
Rho Diabetes	1.000	1.000	**0.222 (0.030)**	**0.005 (0.020)**	0.048 (0.543)	−0.050 (0.545)
Rho Hypertension	**0.222 (0.030)**	**0.005 (0.020)**	1.000	1.000	0.049 (0.552)	0.005 (0.940)
Rho Vitamin D deficiency	0.048 (0.543)	−0.050 (0.545)	0.049 (0.552)	0.005 (0.940)	1.000	1.000

Note: *p* value is reported in the parenthesis, figures in bold are significant at *p* < 0.05

Altering the definition of diabetes (Table 5) did not materially impact results with respect to men. Among women age and central obesity were no longer significant predictors of diabetes. With respect to hypertension, central obesity was no longer a significant determinant among men nor was obesity among women. Vitamin D deficiency results remained unaffected. When medication was added to the analysis (Table 6), as one would expect diabetic medicines are strongly predictive of diabetic status. With respect to other variables with the exception of high income in females which is no longer significant results remain materially the same. With respect to hypertension, with the exception of diabetic medication being predictive of hypertension status in women (but not men) results remains essentially the same as with Table 2. Again vitamin D status results remain unaffected and Rho remains significant between hypertension and diabetes and insignificant with vitamin D.

Variations in the specification of vitamin D deficiency levels are reported in supplement 1 (Tables 7a and 7b) and variations in exposure to sunlight reported in supplement 2 (Tables 8a and 8b). Lowering or raising the vitamin D threshold had no material effect on relationships with respect to diabetes or hypertension though with respect to vitamin D status lowering the threshold did increase the impact of sun exposure while raising it reduced its impact. Similarly, among men the role of covariates related to obesity and milk consumption changed. Lowering or raising the threshold used to define sun exposure similarly had no material effect on relationships with respect to diabetes or hypertension. While raising the threshold had no material effect in respect of vitamin D, sun exposure became insignificant among women at the lower threshold but remained unchanged in men. In each case Rho remained significant between hypertension and diabetes but insignificant with vitamin D status.

## Discussion

The pandemic of ‘diabesity’ presents a growing challenge for healthcare systems globally [2]. Diabetes is related to cardiovascular disease and obesity is associated with a range of conditions including diabetes, cardiovascular disease and certain cancers [26]. Some studies indicate that vitamin D, obesity, diabetes and cardiovascular disease comprise a ‘relationship set’ though the nature of the set of relationships remain unclear and are the subject of ongoing research [9]. In our analysis, we did not find evidence of a direct relationship between hypertension or type 2 diabetes and levels of vitamin D in either men or women. Similarly, we found no evidence of unobserved heterogeneity between vitamin D levels and either type 2 diabetes or hypertension. These findings remained robust over a series of sensitivity analyses in which the range and specification of covariates were altered as were the measurement of outcomes. The findings suggest that while vitamin D deficiency is prevalent in the Saudi population aged 15 and over, there does not appear to be a significant association between diabetes or hypertension risk and vitamin D deficiency, nor is there evidence that might suggest the existence of an indirect relationship related to unobserved heterogeneity. This result supports the suggestion that vitamin D may not offer a useful target by which to reduce risks for hypertension or diabetes [9]. That is, those epidemiological observations of a relationship between vitamin D deficiency, hypertension and diabetes are associative rather than causative [27, 28]. While vitamin D deficiency may because of its association with other conditions such as osteoporosis or osteopenia warrant appropriate interventions it is unlikely these will confer any protective effect in respect of diabetes or hypertension.

Significant relationships between vitamin D status, exposure to sunlight and consumption of milk were observed though only among women thereby suggesting dietary advice aimed at improving vitamin D may benefit this group - a finding echoed in previous work in Saudi Arabia by AlQuaiz et al.^12^. While a relationship between obesity and vitamin D status was found in men (but not women), it does not appear to offer additional information that might be of use in understanding or addressing diabesity in this context. The results in relation to vitamin deficiency are interesting in their own right and provide information that might be of use to public health planners and policy makers. Among women, nutritional advice and advice to encourage greater exposure to sunlight would benefit those who are vitamin D deficient. That age appears unrelated to vitamin D deficiency among women suggests the advice would benefit women of different ages.

In respect of obesity, whether in general or specifically central obesity (waist circumference) these were related to both hypertension and diabetes status in men and women (with the exception of hypertension in women when sedentary behaviour was included as a covariate where it only attained borderline significance.) The strength of the relationship varied between the two genders – appearing broadly stronger in men than women. The findings in respect of sedentary behaviour – significant and positively related to diabetes risk in women and borderline positively significant with respect to hypertension and diabetes in men is also notable. These findings underscore the importance of maintaining a healthy weight to reduce the risk of diabetes and hypertension with a reduction in sedentariness providing both a means of reducing weight and reducing risk independent of weight. The significant positive correlation in errors from these functions though is indicative of unobserved heterogeneity. It suggests that where we under-predict diabetes risk we under-predict the risk of hypertension. This likely reflects the exclusion of relevant covariates related perhaps to unobserved behaviors, duration of the condition or vulnerability to risk exposure. This suggests further research into these relationships is warranted, perhaps using additional data to that which was available here.

With the exception of age, other variables were largely non-significant. Perhaps surprisingly ever having smoked was not related to hypertension. This suggests the usefulness of *ever* having smoked as an indicator of *current* health behaviors and risks is perhaps weaker than one might expect. Given “ever” may include widely different patterns of behavior and the well established links between smoking and health, some caution is warranted in the interpretation of this result.

Our study has a number of limitations. First, our data are cross-sectional in nature and in consequence caution continues to be advisable in drawing inferences with respect to possible causal relationships. Second, while the SHIS offers a rich data source on a large representative sample, in a number of areas the data are self-reported – for example in respect of exercise and diet – while other data – for example in respect of chronic disease, neither the time since onset of these nor their severity are known. Similarly, measurement error and the inability to characterize respondents as fully as one might like are in consequence inevitable for example with respect to medication use. Further research could usefully explore these issues as additional data become available. Whether the relationships observed here extend to those using supplements could usefully be explored in further research as could the stability of relationships over time, through the use of other waves of SHIS.

## Conclusion

The analysis suggests a relationship between obesity and the distribution of body fat with the risk of diabetes and hypertension in men and women in Saudi Arabia. The nature of that relationship varies between men and women. The study indicates the existence of unobserved heterogeneity in the relationships between diabetes and hypertension risk that warrants further investigation. That obesity and sedentary behaviour were both related to risk suggests exercise in maintenance of a healthy weight as potential targets for policy intervention. We found no relationship between vitamin D levels and risk of either diabetes or hypertension. We found no evidence of unobserved heterogeneity in risk related to vitamin D status when examined with diabetes and hypertension. While significant predictors of vitamin D status were identified and offer possible policy targets, there appears no support for the targeting of vitamin D levels per se in reducing the risk of either hypertension or diabetes in this context.

## Supplementary information

**Additional file 1 Table 7a**. Result of multivariable probit model analysis with vitamin D deficiency cut off level at 10 ng/mL (n = 2084). **Table 7b**. Result of multivariable probit model analysis with vitamin D deficiency cut off level at 12 ng/mL (n = 2084). **Table 8a**. Result of multivariable probit model analysis with sun exposure defined at 10 min per day. **Table 8b**. Result of multivariable probit model analysis with sun exposure defined at 30 min per day.

## Data Availability

The data sets analysed during the current study are not publicly available. Any request to access the data needs to be addressed to the Saudi Ministry of Health. Further details including contact details can be obtained at the following link: https://www.moh.gov.sa/en/Ministry/Statistics/Pages/healthinformatics.aspx

## Abbreviations

BMI: Body mass index
SHIS: Saudi Health Interview Survey
SURVBP: Seemingly unrelated bivariate probit
25(OH)D: 25-hydroxyvitamin D
