# Supplementary material for: The relationship between obesity, diabetes, hypertension and vitamin D deficiency among Saudi Arabians aged 15 and over: results from the Saudi health interview survey
Source: BMC Endocr Disord. 2020 Jun 5;20:81. doi: 10.1186/s12902-020-00562-z (PMC7275458; doi:10.1186/s12902-020-00562-z)
Supplement: Supplementary file 1 — Additional file 1 Table 7a. Result of multivariable probit model analysis with vitamin D deficiency cut off level at 10 ng/mL (n = 2084). Table 7b. Result of multivariable probit model analysis with vitamin D deficiency cut off level at 12 ng/mL (n = 2084). Table 8a. Result of multivariable probit model analysis with sun exposure defined at 10 min per day. Table 8b. Result of multivariable probit model analysis with sun exposure defined at 30 min per day. [file 12902_2020_562_MOESM1_ESM.docx]

| Supplement table 7a. Result of multivariable probit model analysis with vitamin D deficiency cut off level at 10 ng/mL (n=2,084) | | | | | | |
| --- | --- | --- | --- | --- | --- | --- |
|  | Diabetes | | Hypertension | | Vitamin D deficiency | |
|  | Female | Male | Female | Male | Female | Male |
| Age | **0.099**  **(0.009)** | **0.147**  **(0.000)** | **0.189**  **(0.000)** | **0.090**  **(0.001)** | **-0.060**  **(0.010)** | 0.003  (0.917) |
| Age^2 | -0.001  (0.077) | **-0.001**  **(0.000)** | **-0.001**  **(0.000)** | -0.000  (0.096) | **0.001**  **(0.022)** | -0.000  (0.824) |
| Obesity | 0.083  (0.572) | 0.221  (0.090) | **0.303**  **(0.033)** | **0.587**  **(0.000)** | 0.333  (0.133) | 0.294  (0.123) |
| Central obesity | **0.597**  **(0.001)** | **0.333**  **(0.008)** | 0.111  (0.501) | **0.287**  **(0.036)** | 0.069  (0.750) | -0.182  (0.358) |
| High income | **0.431**  **(0.037)** | 0.164  (0.256) | -0.353  (0.150) | 0.068  (0.672) | -0.057  (0.868) | 0.137  (0.592) |
| Vitamin D level | 0.004  (0.207) | 0.004  (0.117) | -0.002  (0.656) | 0.003  (0.312) | - | - |
| Ever smoker | - | - | 0.571  (0.267) | -0.094  (0.448) | - | - |
| Milk consumption | - | - | - | - | **-0.570**  **(0.001)** | **3.656**  **(0.000)** |
| Sun exposure | - | - | - | - | **-3.655**  **(0.000)** | 0.128  (0.523) |
| Constant | **-5.260**  **(0.000)** | **-6.098**  **(0.000)** | **-7.156**  **(0.000)** | **-4.973**  **(0.000)** | -0.258  (0.567) | **-5.831**  **(0.000)** |
| Rho Diabetes | 1.000 | 1.000 | **0.272**  **(0.003)** | **0.207**  **(0.005)** | 0.005  (0.970) | 0.183  (0.220) |
| Rho Hypertension | **0.272**  **(0.003)** | **0.207**  **(0.005)** | 1.000 | 1.000 | 0.206  (0.278) | 0.018  (0.873) |
| Rho Vitamin D deficiency | 0.005  (0.970) | 0.183  (0.220) | 0.206  (0.278) | 0.018  (0.873) | 1.000 | 1.000 |
| Note: p value is reported in the parenthesis, figures in bold are significant at p<0.05 | | | | | | |

| Supplement table 7b. Result of multivariable probit model analysis with vitamin D deficiency cut off level at 12 ng/mL (n=2,084) | | | | | | |
| --- | --- | --- | --- | --- | --- | --- |
|  | Diabetes | | Hypertension | | Vitamin D deficiency | |
|  | Female | Male | Female | Male | Female | Male |
| Age | **0.100**  **(0.009)** | **0.147**  **(0.000)** | **0.189**  **(0.000)** | **0.092**  **(0.000)** | **-0.046**  **(0.031)** | 0.004  (0.834) |
| Age^2 | -0.001  (0.077) | **-0.001**  **(0.000)** | **-0.001**  **(0.000)** | -0.000  (0.080) | 0.000  (0.130) | -0.000  (0.660) |
| Obesity | 0.082  (0.577) | 0.220  (0.091) | **0.298**  **(0.035)** | **0.591**  **(0.000)** | 0.052  (0.763) | 0.253  (0.105) |
| Central obesity | **0.597**  **(0.001)** | **0.334**  **(0.008)** | 0.123  (0.456) | **0.285**  **(0.037)** | 0.279  (0.106) | -0.176  (0.295) |
| High income | **0.431**  **(0.037)** | 0.163  (0.258) | -0.354  (0.151) | 0.068  (0.670) | -0.043  (0.870) | 0.016  (0.944) |
| Vitamin D level | 0.004  (0.222) | 0.004  (0.139) | -0.002  (0.518) | 0.003  (0.211) | - | - |
| Ever smoker | - | - | 0.558  (0.276) | -0.088  (0.479) | - | - |
| Milk consumption | - | - | - | - | **-0.485**  **(0.001)** | 0.174  (0.410) |
| Sun exposure | - | - | - | - | -0.742  (0.055) | 0.127  (0.443) |
| Constant | **-5.260**  **(0.000)** | **-6.099**  **(0.000)** | **-7.130**  **(0.000)** | **-5.043**  **(0.000)** | -0.255  (0.530) | **-2.154**  **(0.000)** |
| Rho Diabetes | 1.000 | 1.000 | **0.275**  **(0.002)** | **0.209**  **(0.005)** | -0.015  (0.896) | 0.088  (0.498) |
| Rho Hypertension | **0.275**  **(0.002)** | **0.209**  **(0.005)** | 1.000 | 1.000 | 0.020  (0.897) | 0.150  (0.136) |
| Rho Vitamin D deficiency | -0.015  (0.896) | 0.088  (0.498) | 0.020  (0.897) | 0.150  (0.136) | 1.000 | 1.000 |
| Note: p value is reported in the parenthesis, figures in bold are significant at p<0.05 | | | | | | |

| Supplement table 8a. Result of multivariable probit model analysis with sun exposure defined at 10 minutes per day | | | | | | |
| --- | --- | --- | --- | --- | --- | --- |
|  | Diabetes | | Hypertension | | Vitamin D deficiency | |
|  | Female | Male | Female | Male | Female | Male |
| Age | **0.098**  **(0.009)** | **0.147**  **(0.000)** | **0.188**  **(0.000)** | **0.090**  **(0.001)** | -0.023  (0.070) | -0.014  (0.284) |
| Age^2 | -0.001  (0.079) | **-0.001**  **(0.000)** | **-0.001**  **(0.000)** | -0.000  (0.094) | 0.000  (0.302) | 0.000  (0.881) |
| Obesity | 0.085  (0.563) | 0.217  (0.095) | **0.299**  **(0.035)** | **0.587**  **(0.000)** | 0.086  (0.423) | **0.254**  **(0.022)** |
| Central obesity | **0.602**  **(0.001)** | **0.333**  **(0.008)** | 0.127  (0.441) | **0.287**  **(0.036)** | 0.143  (0.193) | **-0.236**  **(0.045)** |
| High income | **0.436**  **(0.035)** | 0.164  (0.257) | -0.347  (0.160) | 0.069  (0.668) | -0.147  (0.330) | 0.040  (0.771) |
| Vitamin D level | 0.005  (0.133) | 0.004  (0.215) | -0.001  (0.741) | 0.003  (0.321) | - | - |
| Ever smoker | - | - | 0.544  (0.285) | -0.095  (0.448) | - | - |
| Milk consumption | - | - | - | - | **-0.352**  **(0.001)** | -0.023  (0.847) |
| Sun exposure | - | - | - | - | -0.095  (0.375) | 0.086  (0.365) |
| Constant | **-5.261**  **(0.000)** | **-6.065**  **(0.000)** | **-7.150**  **(0.000)** | **-4.978**  **(0.000)** | 0.368  (0.169) | **-0.634**  **(0.025)** |
| Rho Diabetes | 1.000 | 1.000 | **0.273**  **(0.003)** | **0.205**  **(0.006)** | 0.056  (0.463) | -0.012  (0.881) |
| Rho Hypertension | **0.273**  **(0.003)** | **0.205**  **(0.006)** | 1.000 | 1.000 | 0.062  (0.443) | 0.008  (0.913) |
| Rho Vitamin D deficiency | 0.056  (0.463) | -0.012  (0.881) | 0.062  (0.443) | 0.008  (0.913) | 1.000 | 1.000 |
| Note: p value is reported in the parenthesis, figures in bold are significant at p<0.05 | | | | | | |

| Supplement table 8b. Result of multivariable probit model analysis with sun exposure defined at 30 minutes per day | | | | | | |
| --- | --- | --- | --- | --- | --- | --- |
|  | Diabetes | | Hypertension | | Vitamin D deficiency | |
|  | Female | Male | Female | Male | Female | Male |
| Age | **0.098**  **(0.009)** | **0.147**  **(0.000)** | **0.188**  **(0.000)** | **0.090**  **(0.001)** | -0.024  (0.065) | -0.015  (0.260) |
| Age^2 | -0.001  (0.079) | **-0.001**  **(0.000)** | **-0.001**  **(0.000)** | -0.000  (0.094) | 0.000  (0.275) | 0.000  (0.851) |
| Obesity | 0.085  (0.565) | 0.217  (0.095) | **0.298**  **(0.035)** | **0.587**  **(0.000)** | 0.080  (0.452) | **0.247**  **(0.026)** |
| Central obesity | **0.601**  **(0.001)** | **0.333**  **(0.008)** | 0.127  (0.441) | **0.287**  **(0.036)** | 0.134  (0.222) | **-0.234**  **(0.047)** |
| High income | **0.436**  **(0.036)** | 0.164  (0.257) | -0.348  (0.159) | 0.069  (0.668) | -0.109  (0.475) | 0.046  (0.733) |
| Vitamin D level | 0.005  (0.137) | 0.004  (0.220) | -0.001  (0.737) | 0.003  (0.315) | - | - |
| Ever smoker | - | - | 0.545  (0.284) | -0.095  (0.448) | - | - |
| Milk consumption | - | - | - | - | **-0.361**  **(0.001)** | -0.021  (0.859) |
| Sun exposure | - | - | - | - | **-0.373**  **(0.047)** | -0.016  (0.885) |
| Constant | **-5.259**  **(0.000)** | **-6.064**  **(0.000)** | **-7.147**  **(0.000)** | **-4.979**  **(0.000)** | 0.382  (0.152) | **-0.571**  **(0.041)** |
| Rho Diabetes | 1.000 | 1.000 | **0.273**  **(0.003)** | **0.205**  **(0.006)** | 0.053  (0.479) | -0.016  (0.844) |
| Rho Hypertension | **0.273**  **(0.003)** | **0.205**  **(0.006)** | 1.000 | 1.000 | 0.061  (0.457) | 0.011  (0.880) |
| Rho Vitamin D deficiency | 0.053  (0.479) | -0.016  (0.844) | 0.061  (0.457) | 0.011  (0.880) | 1.000 | 1.000 |
| Note: p value is reported in the parenthesis, figures in bold are significant at p<0.05 | | | | | | |
